# The PLSDB 2025 update: enhanced annotations and improved functionality for comprehensive plasmid research

**DOI:** 10.1093/nar/gkae1095

**Published:** 2024-11-20

**Authors:** Leidy-Alejandra G Molano, Pascal Hirsch, Matthias Hannig, Rolf Müller, Andreas Keller

**Affiliations:** Chair for Clinical Bioinformatics, Saarland University, 66123 Saarbrücken, Germany; Chair for Clinical Bioinformatics, Saarland University, 66123 Saarbrücken, Germany; Clinic of Operative Dentistry, Periodontology and Preventive Dentistry, Saarland University, 66421 Homburg, Germany; Helmholtz Institute for Pharmaceutical Research Saarland (HIPS), Helmholtz Centre for Infection Research, 66123 Saarbrücken, Germany; Chair for Clinical Bioinformatics, Saarland University, 66123 Saarbrücken, Germany; Helmholtz Institute for Pharmaceutical Research Saarland (HIPS), Helmholtz Centre for Infection Research, 66123 Saarbrücken, Germany; PharmaScienceHub, 66123 Saarbrücken, Germany

## Abstract

Plasmids are extrachromosomal DNA molecules in bacteria and archaea, playing critical roles in horizontal gene transfer, antibiotic resistance, and pathogenicity. Since its first release in 2018, our database on plasmids, PLSDB, has significantly grown and enhanced its content and scope. From 34 513 records contained in the 2021 version, PLSDB now hosts 72 360 entries. Designed to provide life scientists with convenient access to extensive plasmid data and to support computer scientists by offering curated datasets for artificial intelligence (AI) development, this latest update brings more comprehensive and accurate information for plasmid research, with interactive visualization options. We enriched PLSDB by refining the identification and classification of plasmid host ecosystems and host diseases. Additionally, we incorporated annotations for new functional structures, including protein-coding genes and biosynthetic gene clusters. Further, we enhanced existing annotations, such as antimicrobial resistance genes and mobility typing. To accommodate these improvements and to host the increase plasmid sets, the webserver architecture and underlying data structures of PLSDB have been re-reconstructed, resulting in decreased response times and enhanced visualization of features while ensuring that users have access to a more efficient and user-friendly interface. The latest release of PLSDB is freely accessible at https://www.ccb.uni-saarland.de/plsdb2025.

## Graphical Abstract

**Graphical Abstract:**
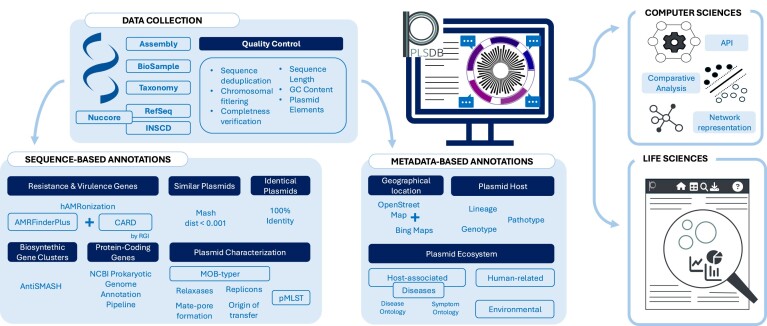

## Introduction

Plasmids are extrachromosomal mobile genetic elements capable of autonomous replication and transfer across host organisms, from narrow to broad host range (1,2). These genetic elements are present across different domains of life but have been extensively studied in bacteria (1–3). It is estimated that approximately 50% of bacteria harbor one or more plasmids, making them a significant source of genetic variability (4). Consequently, plasmids have become a molecular cornerstone in the One Health Era due to their capacity to spread antibiotic resistance and virulence factors through horizontal gene transfer (HGT) events (1,5). Therefore, understanding plasmid sequences has become crucial in microbiome studies, as it allows for the analysis of their association with clinically relevant traits and comprehension of these connections with treatment responses.

In this line, we have developed a database that facilitates easy access to validated plasmids, PLSDB. Our repository has been supporting plasmids research since its inception in 2018, by offering a curated source of plasmids from NCBI (6) and INSDC (7), free from chromosomal and redundant sequences (8,9). Plasmids in PLSDB are presented within a comprehensive framework that includes functional annotation and extensive metadata. This metadata spans nucleotide and assembly sequence information, host taxonomy, geographical and ecological information, similarity to other plasmids, and functional structures. Beyond allowing life scientists convenient access to data collections, one ambition in developing such resources is in facilitating computer scientists to have curated data for artificial intelligence applications. Indeed, PLSDB has also served as a foundation for advanced machine learning models such as geNomad (10) and PLASMe (11), which predict plasmid sequences from metagenomic assemblies. These models have facilitated significant advancements in plasmid research, as exemplified by the IMG/PR database (12). This database provides an extensive repository of 699 973 plasmid sequences identified from diverse microbiome samples, offering a wealth of metadata including geographical, ecological, and functional annotations. While the IMG/PR database excels in breadth and diversity, our PLSDB stands out with its carefully curated content. The complementary strengths of both databases underscore the importance of having robust, diverse resources to advance the field.

To further support plasmid research, we have conducted a major update of PLSDB, incorporating annotations for functional structures and the visualization of features. These updates aim to provide researchers with more comprehensive tools for plasmid analysis, facilitating deeper insights into plasmid dynamics, their role in microbial communities, and their impact on public health through the spread of antibiotic resistance.

## Materials and methods

### Data collection

Records are retrieved from NCBI Nucleotide database (Entrez Direct v.16.2) (INDSC -DDBJ, EMBL/ENA, Genbak, RefSeq) on 31/05/2024 using the following query: ‘biomol_genomic[PROP] AND plasmid[FILT] AND (bacteria[FILT] OR archaea[FILT]) NOT complete cds[TITL] NOT gene[TITL] NOT genes[TITL] NOT contig[TITL] NOT scaffold[TITL] NOT whole genome map[TITL] NOT partial sequence[TITL] NOT (partial[TITL] AND plasmid[TITL]) NOT locus[TITL] NOT region[TITL] NOT fragment[TITL] NOT integron[TITL] NOT transposon[TITL] NOT insertion sequence[TITL] NOT insertion element[TITL] NOT phage[TITL] NOT operon[TITL]’ (13). To broaden the scope of PLSDB we have incorporated the Archaea kingdom into our data collection query (8). Document summary was fetched for each hit and subsequently linked to their correspondent Biosample and Assembly record, when available. The retrieval of BioSample attributes has been improved by homogenizing the attribute's name according to the BioSample Package guidelines provided by NCBI (https://www.ncbi.nlm.nih.gov/biosample/docs/attributes/). The current list of BioSample attributes incorporated into PLSDB can be found in https://www.ccb.uni-saarland.de/plsdb2025/static/biosample_attributes_plsdb.csv. Finally, measures have been implemented to exclude anomalous assemblies by systematically filtering out assemblies tagged as ‘anomalous’.

### Deduplication

Identical sequence groups are identified using seqkit2 (14) (version 2.8.1; cla: ‘seqkit rmdup –by-seq‘). To ensure the inclusion of the most informative data while minimizing redundancy, for each group of identical sequences one record is selected based on the following criteria: (i) preference for RefSeq (15) records: prioritizing RefSeq records over those from INSDC repository; (ii) enriched metadata information: records enriched with supplementary details such as geographical location, BioSample and Assembly information, are favoured. In addition, records with more recent assembly release date, nucleotide creation date, and highest coverage, are preferred. Despite the selection of a singular record for database inclusion, the associated information pertaining to their identical sequence mates is retained, preserving contextual information of biological relevance.

### Filtering of chromosomal sequences

To remove non-plasmids or non-complete plasmid sequences, descriptions were scanned as previously described (8). If assembly information was available, only records with the ‘completness’, ‘lastest’ and ‘non-anomalous’ assembly status were retained. If no completeness tag was associated to the record, then only the assembly tag was used and vice versa. Only the non-empty tags were used to remove the records.

For the identification of chromosomal sequences incorrectly identified as plasmids, putative chromosomal sequences are listed by performing a *in silico* rMLST (16) analysis (i.e searching the 53 *rps* genes) using Mash distances (17) (version 2.3, cla: ‘mash sketch –S 123 –k 21 –s 1000 –i’; ‘mash dist –d 0.00123693’) and verified with BLAST (18) as previously described (8). Candidates for further screen are considered if more than 5 unique *rps* genes are detected in the sequence. Subsequently, they are compared against a local chromosomal version of NCBI nucleotide database (6) using Mash distances and Blast verification. The local chromosomal dataset was retrieved using the query: ‘(Bacteria OR Archaea) NOT plasmid [FILT] NOT complete cds[TITL] NOT gene[TITL] NOT genes[TITL] NOT contig[TITL] NOT scaffold[TITL] NOT whole genome map[TITL] NOT partial sequence[TITL] NOT locus[TITL] NOT region[TITL] NOT fragment[TITL] NOT integron[TITL] NOT transposon[TITL] NOT insertion sequence[TITL] NOT insertion element[TITL] NOT phage[TITL] NOT operon[TITL] NOT whole genome shotgun[TITL] NOT assembly[TITL]’. Data available until 31/05/2024 date was included. Plasmid candidates with at least one hit in the chromosomal dataset with at least 99% identity and 80% query coverage were considered as chromosomal sequences and excluded from the plasmid collection (version 2.15.0; cla ‘blastn -task megablast -perc_identity 99 -qcov_hsp_perc 80 -evalue 0.05 -max_target_seqs 10 -max_hsp 10’).

To further detect and discard biologically implausible plasmids, plasmids with outlier's values of GC content or log10 sequence length were submitted to further manual inspection. Values were considered outliers according to the interquartile range criterion (value < Q_0.25_–1.5*IQR or value > Q_0.75_ + 1.5*IQR). Candidate anomalous plasmids with a total absence of plasmid typing (including replicon, relaxases, mate pore formatting, origin of transfer, and pMLST) and total absence of annotated genetic elements (i.e. genes, insertion sequences) were suppressed. Additionally, candidate anomalous plasmids with a sequence length <1 kb or length >4 MB were further suppressed.

### Ecosystem and disease identification

Each biosample record linked to a plasmid entry proves rich source of information and is automatically scanned for the identification of potential host-associated disease and ecosystem, as well as environmental-related ecosystems. Ecosystem identification now includes environmental-related habitats and is inferred from the following BioSample attributes: host, host_taxid, host_common_name, host_animal_breed, animal_env, local_class, soil_type, metagenome_source, samp_mat_type, source_type, host_tissue_sampled, tissue, host_body_habitat, isolation_source, env_medium, env_broad_scale and env_local_scale. Host-associated ecosystems are determinate utilizing NCBI Taxonomy (19) and ETE4 (20) (version 4.1.0-beta). Diseases were classified according to Disease and Symptom Ontologies (21,22). Following automatic classification, identified diseases and ecosystems undergo manual curation to validate and refine the classification process. In cases where a sample was associated with multiple habitats and diseases, all the corresponding habitats and diseases were considered.

### Geographical information

Geographical information for plasmid collection was retrieved from BioSample location name (geo_loc_name) or coordinates (lat_lon) if available. When both attributes were provided, coordinates were preferred. Attributes were processed using the Geopy API of OpenStreetMap (planet dump from https://planet.osm.org) and further compared with Bing Maps (accessed 10 July 2024, used under Microsoft Bing Maps Platform APIs’ Terms of Use). Manual inspection was performed when discrepancies between OpenStreetMap and Bing Maps arise.

### Features annotation

Protein-coding genes annotations were retrieved from the automatic NCBI Prokaryotic Genome Annotation Pipeline (PGAP) (23). Antimicrobial Resistance genes annotations were predicted by combining results from AMRFinderPlus (24) (version 3.12.8, database version 2024-01-31.1, cla: ‘amrfinder –report_all_equal –plus –ident_min 0.95 –coverage_min 90’) and RGI (25) (version 6.0.3, CARD version 3.2.9, cla:’rgi main –local –clean –low_quality –include_nudge’) through hAMRonization (26) (version 1.1.4). To avoid redundancy, records sharing the same gene symbol, strand, and genomic coordinates (range +-10nt) were considered duplicates and only AMRFinderPlus records were retained. Biosynthethic Gene Cluster (BGCs) annotations were predicted with antiSMASH (27) (version 7.1.0; cla: ‘antismash –genefinding-tool = prodigal-m’).

### Webserver

For the improvement of the PLSDB webserver, we set up a PostgreSQL database (https://www.postgresql.org/) that can be browsed using DataTables (https://datatables.net/). Plasmid visualization is using CGView.js (28) (https://js.cgview.ca) whereas the ecosystem network visualization is using cytoscape.js (29) (https://js.cytoscape.org/).

## Results

### Description and key content of PLSDB 2025

**Figure 1.**
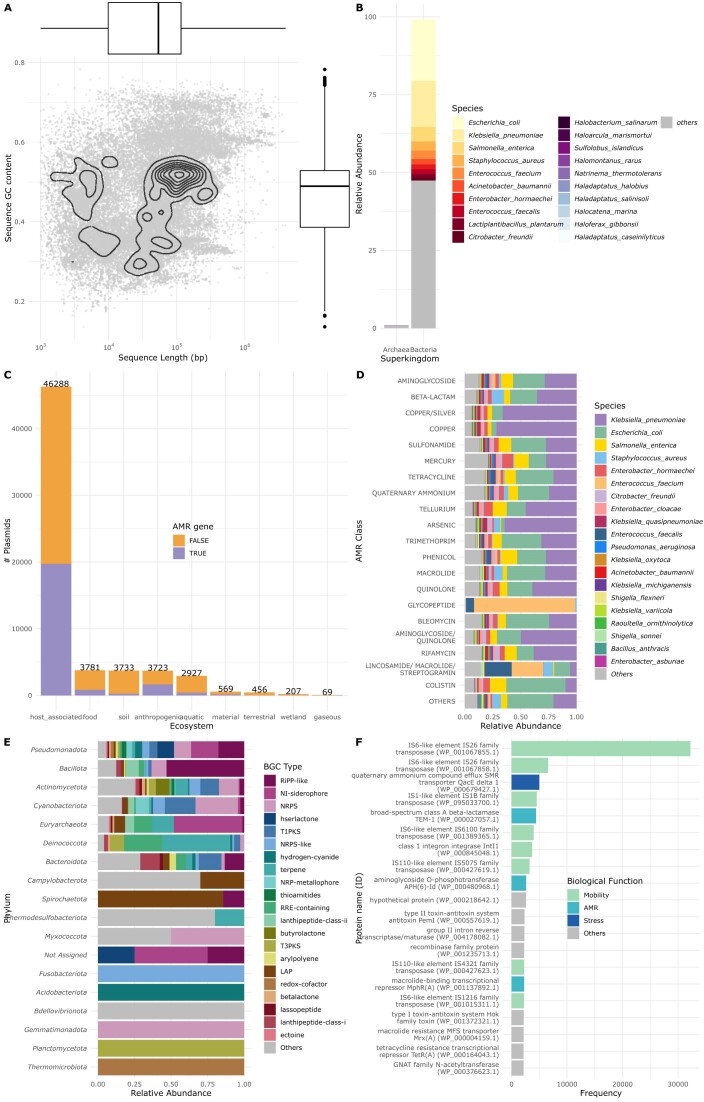
(**A**). Distribution of plasmid sequence length (bp) and GC content. (**B**) Host plasmid distribution at species level. The 10 most represented species are displayed for each kingdom. (**C**) Plasmid ecosystem isolation source distribution. Ecological information from non-repetitive and duplicated plasmids is included. For each ecosystem, the presence of Antimicrobial Resistance (AMR) genes is displayed. (**D**) Plasmid predicted AMR classes distributed by plasmid host specie. Only labels are displayed for the 20 most frequent AMR classes and species. Both AMR classes axis and species legend are ordered by decreasing frequency. (**E**) Plasmid predicted Biosynthetic Gene Cluster (BGC) Types distributed by plasmid host phyla. Only labels are displayed for the 21 most abundant BGC Types (90% abundance). Both phylum axis and BGC Type legend are ordered by decreasing frequency. (**F**) 20 most frequent plasmid proteins coloured by biological function. Protein Identifiers (ID) are from NCBI protein database.

The PLSDB (v. 2024_05_31_v2) contains a total of 72 360 dereplicated plasmids, with 7GB of sequence information, representing a 110% increase versus 2021 version. Records presented a median length of 54kbp and a GC content of 49% (Figure 1A). Notably, 91.7% of these plasmids are annotated as circular.

The host plasmid distribution was predominated by bacterial hosts (71 753), with the most represented phylas being *Pseudomonata* (70.1%), *Bacillota* (19.8%), *Actinomycetota* (3.4%), and *Spirochaetota* (1.8%). Archaeal hosts included 607 plasmids, primarily from *Euryarchaeota* (94.4%) and *Thermoproteota* (4.4%). A total of 4 787 different species had plasmid representation, with *Escherichia coli* being the most abundant (19.7%), followed by *Klebsiella pneumoniae* (14.8%), *Salmonella enterica* (4.7%) and *Staphylococcus aureus* (2.9%) (Figure 1B).

Host-associated ecosystems were the main source of plasmid isolation, accounting for 75% of the abundance. Within these ecosystems, *Homo sapiens* (45%), *Sus scrofa* (3.8%), *Gallus gallus* (3%) and *Bos taurus* (2.6%) were the most frequent host species (Figure 1C). Antimicrobial resistance (AMR) genes were found across all ecosystems, with highest percentage in anthropogenic (40%) and host-associated (35%) ecosystems (Figure 1C). The most frequent AMR gene class was aminoglycoside (14.2%), followed by beta-lactamases (12.6%), and cooper (7.7%) resistance genes (Figure 1D).

Biosynthetic Gene Clusters (BGCs) were predominantly predicted in the *Pseudomonata* phylum (Figure 1E). They mainly contained RiPP-like, NI-siderophore and NRPS classes. Lastly, we identified that the most common proteins in plasmids were related to mobility and AMR genes (Figure 1F).

**Figure 2.**
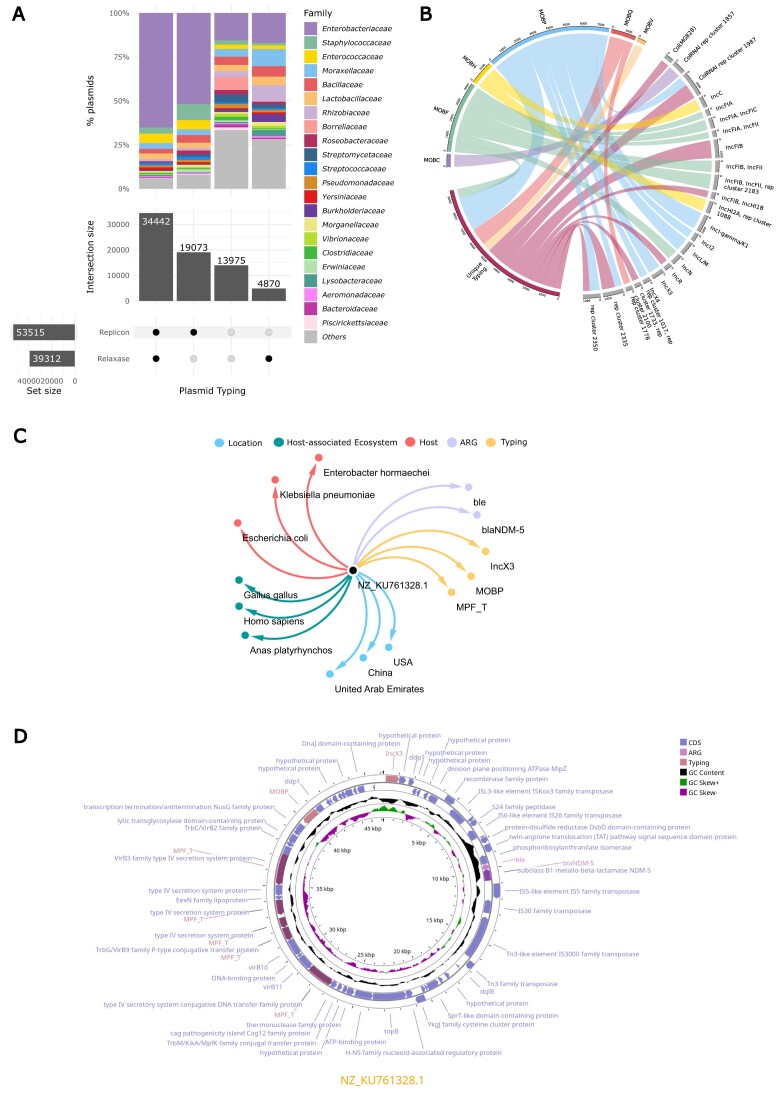
(**A**) Upset plot displaying plasmid typing combinations across PLSDB v.2024_05_31_v2. For each typing combination, the most prevalent plasmid host families are displayed. (**B**) Chord diagram illustrating associations between replicon and relaxase types. Only the 50 most frequent associations are displayed. (**C**) Network representation of NZ_KU761328.1 plasmid characteristics, including geographical locations, host-associated ecosystems, plasmid hosts, typing, and detected antimicrobial resistance genes. (**D**) Sequence visualization of NZ_KU761328.1 plasmid, displaying annotated elements.

Out of the total 72 360 plasmids analyzed, 80.7% were successfully typed based on either replicon or relaxase elements (Figure 2A). Of these, 26.4% were classified solely by replicon elements, 6.7% were categorized only by relaxase elements, and 47.6% were identified through both replicon and relaxase typing methods. Remarkably, archaeal plasmids posed a unique challenge, with only 19 out of 607 (3.1%) being typed by either replicon or relaxase elements. Among the remaining 13 875 plasmids that could not be typed using replicon or relaxase elements, the most common host families were *Enterobacteriaceae* (15.6%), *Borreliaceae* (7.6%) and *Bacillaceae* (4.7%).

Plasmid typing frequency was predominated by MOBP relaxase family (Figure 2B), representing 3.8% of all plasmids. This was followed by IncFIB plasmids at 1.9%, MOBP-IncI-gamma/K1 at 1.9%, and MOBQ at 1.8%.

### Case study

PLSDB is frequently used to screen for potential plasmid candidates or to identify the novelty of experimentally validated ones (30,31) but can also be used to explore the state-of-art of plasmids epidemiology (5). For demonstration purposes, we will investigate plasmids with *Enterobacterales* hosts, pathogens identified as critical priority group by the WHO (32). For the analysis, we will use the renovated browser tables, specifically the Summary table, filtering for plasmids in *Enterobacterales* within the 40–50 kb range that are rich in annotations, including AMR genes and mobility (MOB) typing, applying the ‘not empty’ filter. Further refinement included selecting plasmids with identical sequences (‘NUCCORE has identical’), which filter for plasmids with detected identical records. Among the hits, we will select the plasmid NZ_KU761328.1 to illustrate the enhanced functionalities and graphical output of PLSDB 2025.

The taxonomical data of NZ_KU761328.1 and its identical sequences allowed us to identify the prevalence of this plasmid among a variety of *Enterobacterales* hosts, including *Escherichia coli, Klebsiella pneumoniae* and *Enterobacter hormaechei* (Figure 2C). Moreover, the ecosystem data highlighted the plasmid's versatility, as it is found in multiple host-associated environments, including humans, chickens, and ducks. This broad host range indicates its adaptability and potential to cross species barriers, a critical trait that enhances its role in the dissemination of antimicrobial resistance. Geographic data further underscore its global distribution, with isolated collected from diverse locations such as the United Arab Emirates, China, and the USA.

Plasmid typing by MOB-typer detected the presence of IncX3 replicon and MOBP relaxases. A closer examination of the plasmid's annotations revealed the presence of key AMR genes, particularly *blaNDM-5* and *ble*. The *blaNDM-5* gene is especially concerning as it confers resistance to carbapenems, a class of last-resort antibiotics for many severe bacterial infections. The *ble* gene further complicates the scenario by providing resistance to bleomycin, an antibiotic utilized as chemotherapy agent. Furthermore, MOB typing further identify mating-pair formating T system, suggesting a high potential for horizontal gene transfer via the conjugation apparattus, enhancing the plasmid's ability to spread AMR genes among bacterial populations.

Overall, NZ_KU761328.1 exemplifies the complex challenges posed by plasmids in the global spread of antimicrobial resistance. PLSDB 2025 aims to support the study of such elements by providing detailed data on the presence of virulence and AMR factors, host adaptability, geographical distribution, and mobility capabilities.

## Discussion

With PLSDB, we have developed a resource that serves as a cornerstone for the analysis and understanding of plasmids within bacterial isolates and communities. Since its first release in 2018, PLSDB has significantly expanded and improved, evolving from an initial 13 789 records to now hosting 72 360 entries in this latest 2025 release. The database is widely used, attracting approximately 20 000 users annually, which highlights its importance and utility in the scientific community. This growth reflects our commitment to continuously update and enhance the database, integrating community feedback to improve functionality, performance, and data quality. The latest version of PLSDB includes refined identification and classification of plasmid host ecosystems and host diseases, enriched annotations for new functional structures such as protein-coding genes, biosynthetic gene clusters, and conjugation elements, and improved existing annotations for antimicrobial resistance genes and mobility typing. These enhancements ensure that researchers have access to the most comprehensive and accurate plasmid data available. Another development in the latest release of PLSDB, as for other databases and web services we develop, is the adoption of a year-based versioning system. Instead of using traditional numerical versions, we now use the year of the respective update as the version identifier. This change is intended to make users more aware of the most current versions, ensuring they always have access to the latest data and tools. By adopting this system, we aim to improve citation accuracy and encourage the use of the most up-to-date version of PLSDB, thereby maintaining its relevance and accuracy in ongoing and future research.

Besides PLSDB, other resources for plasmids are emerging. One example is the IMG/PR database that provides a vast and diverse repository of 699 973 plasmid sequences derived from genomes, metagenomes, and metatranscriptomes. Using the microbiome systematically as a source for plasmids along with offering rich metadata makes this database a comprehensive tool for researchers needing access to extensive plasmid data, e.g. for broader ecological studies. This broadness comes at the price of a lower degree of manual curation. Finally, the availability of complementary databases such as IMG/PR and PLSDB ensures that researchers have access to both extensive data diversity and high-quality curated datasets, facilitating a more thorough and nuanced understanding of plasmid dynamics, their role in microbial communities, and their impact on human health and biotechnology.

It is our ambition to continually enhance and refine PLSDB, and the user feedback plays a crucial role in this process. We greatly value the insights and suggestions we receive through various channels, including our GitHub repository (https://github.com/CCB-SB/plsdb/), email, and personal interaction at conferences. We actively use this feedback to guide our updates and improvements. We are committed to addressing such requests from the life science and computer science domains to continuously improve PLSDB.

## Data Availability

The PLSDB webserver is freely accessible at https://www.ccb.uni-saarland.de/plsdb2025. The Python package for API access is available on PLSDBapi GitHub (https://doi.org/10.6084/m9.figshare.27284961.v2). The data collection pipeline can be found on PLSDB GitHub (https://doi.org/10.6084/m9.figshare.27283893) where we are also welcoming any user feedback. The underlaying data of PLSDB has been divided into several tables for dedicated information: Records, Identical Records, Similar Records, BioSample, Assembly, Taxonomy, Plasmid Host Diseases, Plasmid Typing, Antimicrobial Resistance, Biosynthethic Gene Clusters and Plasmid Annotated Elements. The tables are interconnected by unique identifiers such as NUCCORE_ACC, BIOSAMPLE_UID, ASSEMBLY_UID, TAXONOMY_UID and DISEASE_ontid. Data can be downloaded at https://www.ccb.uni-saarland.de/plsdb2025/download.
